# Influence of programmed death ligand 1 (PD-L1) knockout on gut microbiota in experimental autoimmune uveitis

**DOI:** 10.3389/fimmu.2025.1600673

**Published:** 2025-05-20

**Authors:** Junxiang Gu, Yixian Ma, Qing Chang, Ling Chen

**Affiliations:** ^1^ Eye Institute and Department of Ophthalmology, Eye & ENT Hospital, Fudan University, Shanghai, China; ^2^ Shanghai Key Laboratory of Visual Impairment and Restoration, Shanghai, China; ^3^ NHC Key Laboratory of Myopia and Related Eye Diseases; Key Laboratory of Myopia and Related Eye Diseases, Chinese Academy of Medical Sciences, Shanghai, China

**Keywords:** programmed death ligand 1 (PD-L1), gut microbiota, metagenomic analysis, autoimmunity, experimental autoimmune uveitis

## Abstract

**Purpose:**

Programmed death ligand 1 (PD-L1) is a potential target for autoimmune disease therapies. The gut microbiota plays a critical role in autoimmunity, and may influence therapeutic outcomes of immune therapies in cancer. However, the relationship between PD-L1 and gut microbiota in autoimmune conditions remains unclear. This study aims to investigate the effect of PD-L1 knockout on gut microbiota in an experimental autoimmune uveitis (EAU) model.

**Methods:**

EAU was induced via immunization with interphotoreceptor retinoid-binding protein peptide 651-670 (IRBP651-670) in either wild type (WT) or PD-L1 knockout (KO) C57BL/6J female mice. Sham adjuvant was administered to WT or PD-L1 KO mice as healthy controls. The severity of EAU was evaluated through clinical evaluation and histopathological gradings. The characteristics of gut microbiota was analyzed using metagenomic sequencing.

**Results:**

Each group consisted of three biological replicates. The clinical and histopathological scores of EAU were significantly higher in KO_EAU mice than in WT_EAU mice. WT_EAU mice exhibited lower microbial richness than their healthy controls (WT mice), while PD-L1 KO in EAU mice (KO_EAU group) led to increased richness when compared to wild type EAU mice (WT_EAU group). EAU induced a reduction in the abundance of *Akkermansia muciniphila A* and an increased in *CAG-485* sp*002362485*. PD-L1 knockout in EAU led to an increased abundance of families Bacteroidaceae, Lachnospiraceae and Ruminococcaceae. EAU was associated with declining microbial tryptophan metabolism and up-regulated functions related to lipid and carbohydrate metabolism; PD-L1 knockout in EAU further increased the metabolism of glycan and biosynthesis of 3-deoxy-α-D-manno-2-octulosonate (Kdo), a key component of bacterial lipopolysaccharide (LPS).

**Conclusion:**

Both EAU and PD-L1 knockout modulate gut microbiota, affecting microbial composition - particularly *Akkermansia*, *CAG-485*, Bacteroidaceae, Lachnospiraceae and Ruminococcaceae - and microbial functions such as lipid, carbohydrate and glycan metabolism.

## 1 Introduction

Autoimmune uveitis is a non-infectious inflammatory disease that primarily affects the uvea, vitreous and retina (1, 2), and is one of the leading causes of irreversible visual loss (3, 4). The pathogenesis of autoimmune uveitis is closely related with immune system dysregulation. CD4+ T cells, especially regulatory T cells (Treg), T helper (Th) 1 and Th17 cells (5), play a central role in the pathogenesis of uveitis, as well as its experimental model, experimental autoimmune uveitis (EAU). Additionally, genetic and environmental factors might contribute to the disease’s onset and progression (6, 7).

Emerging studies have highlighted the significant role of gut microbiota in autoimmune diseases (8–10). In experimental autoimmune encephalomyelitis (EAE), gut microbiota has been shown to influence neuroinflammation by modulating gut dendritic cells and subsequently regulating Tregs and Th17 cells (11). The dysbiosis of gut microbiome could lead to changes in metabolite production, including fatty acids, amino acids and bile acids, all of which have the potential to influence autoimmune responses (12, 13).

Programmed death ligand 1 (PD-L1) is a ligand for the programmed death 1 (PD-1) receptor which is expressed on the surface of lymphocytes and myeloid cells (14). PD-1/PD-L1 signaling pathway plays a crucial role in maintaining immune tolerance by restraining T cells activation (15). Dysfunction of this pathway has been linked to the exacerbation of autoimmune diseases (16, 17). In the context of EAU, Tregs has been shown to suppress effector T cells in a PD-1/PD-L1-dependent manner, suggesting that targeting PD-L1/PD-1 could be a promising therapeutic strategy for uveitis (18).

Several metagenomic studies indicated that gut microbiota could interact with PD-L1/PD-1 signaling and potentially modulate the efficacy of PD-L1 related immune therapies in cancer (19, 20). However, the potential interaction between PD-L1 and gut microbiome in autoimmune uveitis remains poorly understood.

In this study, we aimed to evaluated the impact of PD-L1 on gut microbiota in the EAU model using metagenomic analyses of fecal microbiome. Our goal was to explore the potential role of gut microbiota in modulating the therapeutic effects of PD-L1 targeting in autoimmune uveitis and identify possible biomarkers for PD-L1-based treatment in this context.

## 2 Materials and methods

### 2.1 Establishment and evaluation of EAU model

Specific-pathogen-free (SPF) Wild-type (WT) and PD-L1 knockout (KO) female C57/BL6J mice aged 6–8 weeks were purchased from the Shanghai Model Organisms Center (Shanghai, China) and Cyagen Biosciences (Suzhou, China), respectively. The mice were housed in a SPF environment at 21 ± 1 °C and 55 ± 5% humidity, with a 12-hour light/dark cycle and free access to water and food of the same source. The mice were acclimated for one week before immunization and then kept housed in the same condition for another two weeks after immunization to eliminate possible influences of animal suppliers. The experiments were conducted in accordance with the guide for the care and use of laboratory animals of Fudan University.

The emulsion for immunization was prepared from interphotoreceptor retinoid-binding protein (IRBP) peptide 651-670 (IRBP651-670) (Sangon Biotech, Shanghai, China) solution (4 mg/ml) and complete Freund’s adjuvant (CFA; Sigma-Aldrich Inc., MO, USA) with *Mycobacterium tuberculosis* strain H37Ra (final concentration 2.5 mg/mL; Becton, Dickinson and Company, NJ, USA) at a volume ratio of 1:1. For each mouse, 200 μL of the emulsion was injected subcutaneously at the bilateral base of the tail and flank (50 μL per site). Emulsion without IRBP651–670 was injected to either WT or PD-L1 KO mice as control. 1 μg pertussis toxin (List Labs, CA, USA) resolved in phosphate buffered saline (Thermo Fisher Scientific Inc., MA, USA) was injected intraperitoneally on day 0 and day 2 after immunization.

WT control mice and PD-L1 KO control mice underwent immunization with sham emulsion and were denoted as WT group and KO group, respectively; WT mice and PD-L1 KO mice receiving immunization with IRBP were denoted as WT_EAU group and KO_EAU group, respectively. In each group, the corresponding intervention was performed in 3 independent mice to serve as 3 biological replicates.

14 days post-immunization, dilated fundus photography was performed after anesthesia with 1.25% bromethol (0.2 mL/10g body weight administered intraperitoneally; Aibei Biotechnology Co., Ltd., Nanjing, China). After euthanasia via cervical dislocation under anesthesia with bromethol (same as above), eyes were removed for histopathology; fecal samples were collected from the ileocecal junction for metagenomic sequencing. Clinical scores and histological scores of EAU were graded through the fundus images and Hematoxylin and Eosin stained histological sections of the eye, respectively (21). Grading was conducted in a masked manner by the same researcher.

### 2.2 Gut microbe sequencing

Total microbial DNA was isolated using the OMEGA Mag-Bind Soil DNA Kit (M5635-02; Omega Bio-Tek, GA, USA). The quality control for extracted DNA was performed using Qubit™ 4 Fluorometer (Thermo Fisher Scientific Inc., MA, USA). The extracted DNA was processed to construct metagenome shotgun sequencing libraries with insert sizes of ~400 bp by using Illumina TruSeq Nano DNA LT Library Preparation Kit (Illumina, CA, USA). Sequencing was performed using Illumina NovaSeq platform (Illumina, CA, USA) with PE150 strategy.

### 2.3 Data analysis and statics

Adaptor sequences, duplicated sequences and host sequences were removed from the raw sequencing reads using Cutadapt (version 1.2.1), fastp (version 0.23.2) and Minimap2 (version 2.24-r1122), respectively. Taxonomical classifications of the reads were performed using Kaiju (version 1.9.0) against databases including NCBI-nt, GTDB and RVDB; reads assigned to metazoans or viridiplantae were removed in subsequent analyses. Megahit (version 1.1.2) was used for assembly of the reads using the meta-large preset parameters. Generated contigs (longer than 300 bp) were pooled together and clustered using MMseqs (version 13.45111). Genes in the contigs were predicted using Prodigal (version 2.6.3). The abundances of genes were accessed by mapping high-quality reads onto the predicted gene sequences using Minimap2. The functionality of the non-redundant genes was obtained by annotation using MMseqs2 against KEGG database and using Diamond (version 2.0.15) against MetaCyc database.

The statistics were conducted using SPSS version 22.0 (SPSS Inc., Chicago, IL, USA) for experimental data and R software version 3.6.1 for metagenome analyses. *Post-hoc* power (1-β) was calculated using the POWER procedure of SAS V9.4 (SAS Institute Inc., NC, USA). The normality was tested using Kolmogorov-Smirnov test. Normally-distributed continuous variables were represented in the form of mean ± standard deviation, while other variables were represented as median with range. Mann-Whitney U test was used to compare the scores of EAU. Beta diversity analysis was performed to investigate the compositional variation of microbe using Bray-Curtis distance metric and visualized via principal coordinates analysis (PCoA) and nonmetric multidimensional scaling (NMDS). Alpha diversity metrics including Chao1 index, observed species, Shannon index and Simpson index were calculated and compared using Kruskal-Wallis test and Dunn’s test using the vegan package and visualized via ggplot2 package. Hierarchical clustering was conducted using the vegan, ape and ggtree package. LEfSe (linear discriminant analysis [LDA] effect size) was performed to detect differentially abundant taxa and functions using the LEfSe package in Python. The *P* values < 0.05 were considered statistically significant.

## 3 Results

### 3.1 Severity of EAU

**Figure 1:**
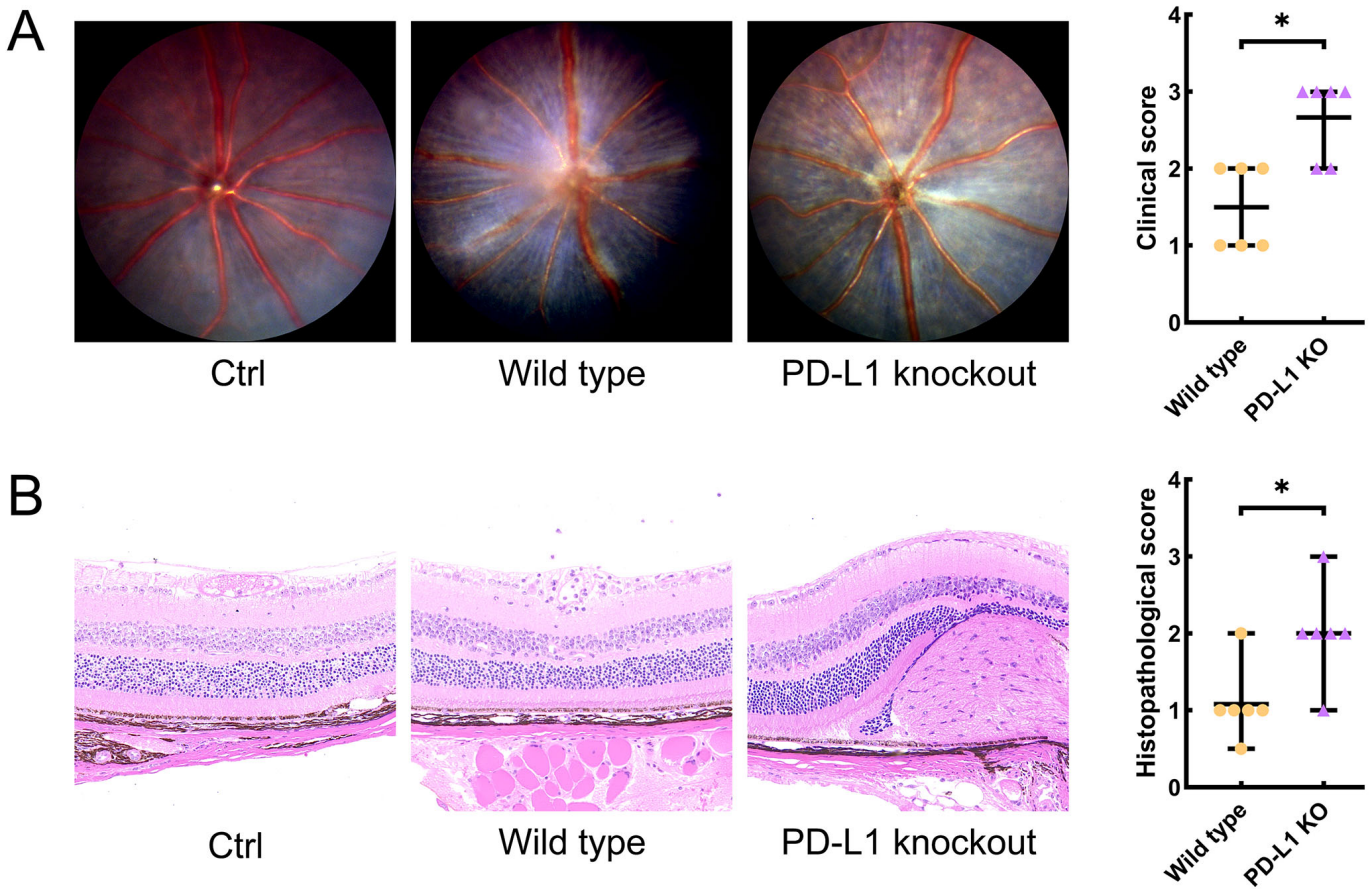
Clinical scores and histological scores of EAU were graded through the dilated fundus photography **(A)** and Hematoxylin and Eosin stained histological sections of the eye **(B)** on day 14 after immunization, respectively. Fundus images and histopathological sections of the eye indicated that PD-L1 knockout could lead to aggravated retinal inflammation, photoreceptor damage and granulomas, as well as the increase in the clinical and histopathological scores of EAU mice. Ctrl, control; PD-L1, programmed death ligand 1; KO, knockout; EAU, experimental autoimmune uveitis; * P<0.05.

No clinical or histopathological sign of EAU was observed in the WT group or KO group. The median grades (range) for clinical and pathological scoring of the WT_EAU group were 2 (1-2) and 1 (0.5-2), respectively; those of the KO_EAU group were 3 (2-3) and 2 (1-3), respectively. Both the clinical and histopathological grades of the KO_EAU group were higher than those of the WT_EAU group (Mann-Whitney U test; P=0.026 [1-β=0.664] and P=0.041 [1-β=0.533], respectively) (**Figure 1**).

### 3.2 Metagenomic sequencing data analysis for gut microbiome

**Figure 2:**
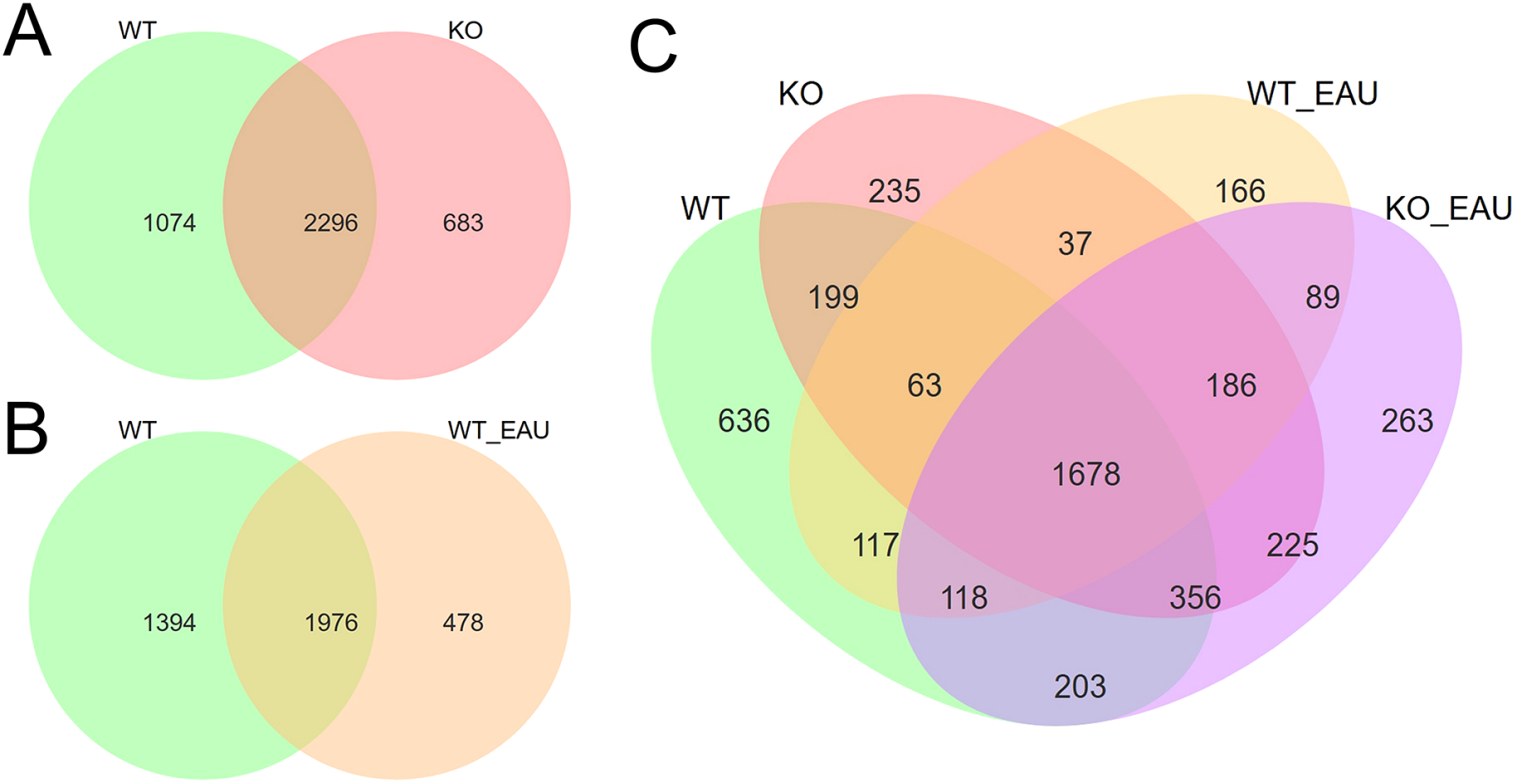
Venn diagrams showed the overlap and difference of gut microbiota in genus level between WT group and KO group **(A)**, between WT group and WT_EAU group **(B)**, as well as among all the 4 groups **(C)**. WT group, wild type control group; KO group, PD-L1 knockout group; WT_EAU group, group of EAU induced in wild type mice; KO_EAU group, group of EAU induced in PD-L1 knockout mice.

Comparison of microbiota composition among the 4 groups revealed the alteration of gut microbiota correlated with PD-L1 KO and EAU (**Figure 2**). When compared to the WT group, KO group had 683 newly detected genera (**Figure 2A**), while EAU group had 478 emerging genera (**Figure 2B**); PD-L1 KO in EAU mice might lead to the increase of 352 genera when all the 4 groups were compared together (**Figure 2C**).

**Figure 3:**
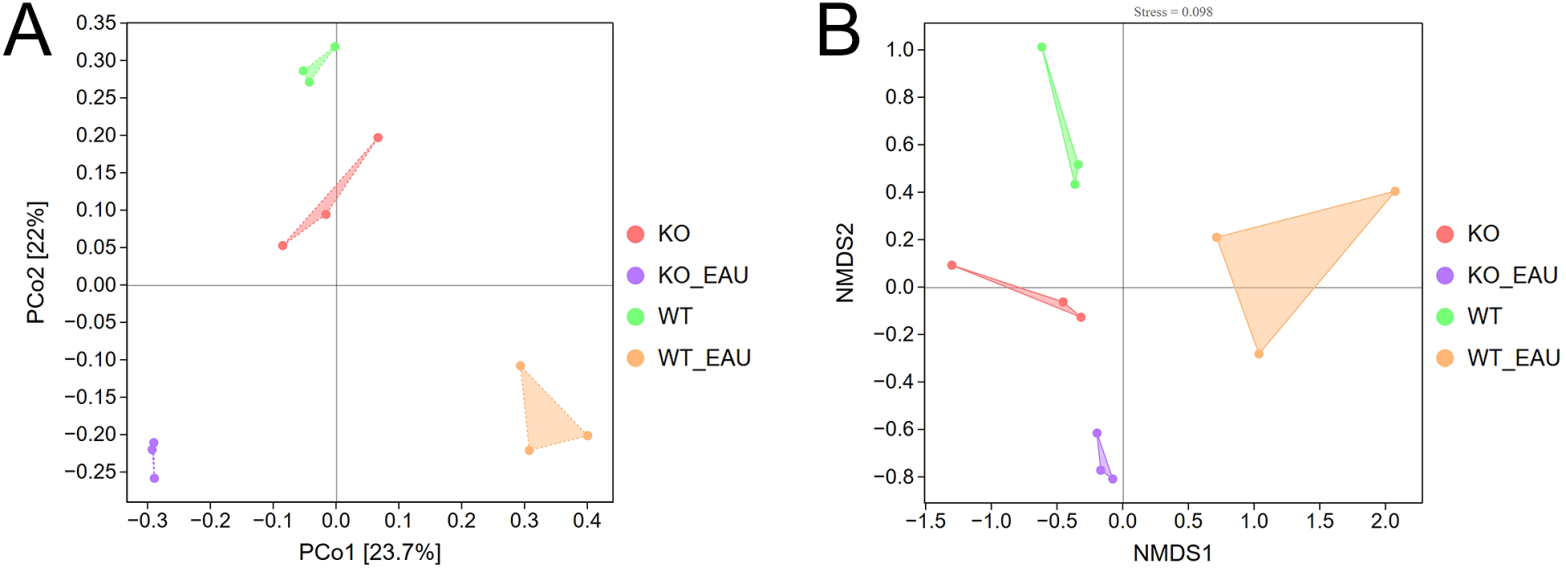
Beta diversity analyses using PCoA **(A)** and NMDS **(B)** indicated sound within-group homogeneity and out-group discrimination of the 4 groups. PCoA, principal coordinates analysis; NMDS, nonmetric multidimensional scaling; WT group, wild type control group; KO group, PD-L1 knockout group; WT_EAU group, group of EAU induced in wild type mice; KO_EAU group, group of EAU induced in PD-L1 knockout mice.

**Figure 4:**
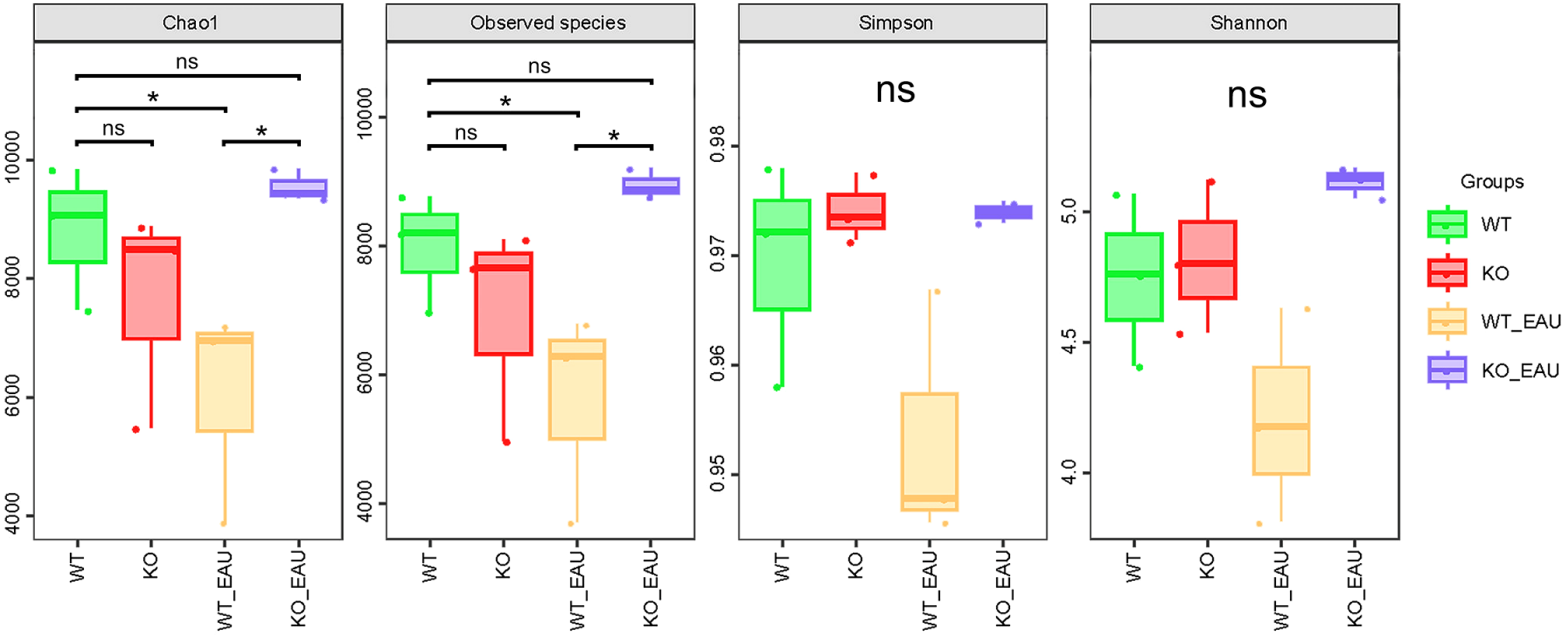
The box plot showed the alpha diversity metrics of the 4 groups. Chao1 index and observed species indicated that the induction of EAU could lead to the decreased richness of gut microbiota, while PD-L1 knockout in EAU mice could lead to the increase in richness when compared to wild type EAU mice. WT group, wild type control group; KO group, PD-L1 knockout group; WT_EAU group, group of EAU induced in wild type mice; KO_EAU group, group of EAU induced in PD-L1 knockout mice; * P<0.05; ns, no significance.

Beta diversity analyses using PCoA (**Figure 3A**) and NMDS (**Figure 3B**) indicated that the within-group homogeneity and out-group discrimination of the 4 groups were relatively satisfactory. Comparisons of alpha diversity metrics showed that PD-L1 KO and induction of EAU could lead to alteration in the richness of gut microbiota. The differences in Chao1 index and observed species were of statistical significance (Kruskal-Wallis test; P=0.049 [1-β=0.348] and P=0.014 [1-β=0.716], respectively); the induction of EAU could lead to the decrease in richness of microbiota (WT versus WT_EAU; P=0.049 [1-β=0.590] and P=0.049 [1-β=0.592] for Chao1 index and observed species, respectively), while knockout of PD-L1 in EAU mice increased the richness (WT_EAU versus KO_EAU; P=0.049 [1-β=0.746] and P=0.049 [1-β=0.757] for Chao1 index and observed species, respectively). No difference in microbiota diversity (reflected by Shannon index and Simpson index) was observed among these groups (**Figure 4**).

**Figure 5:**
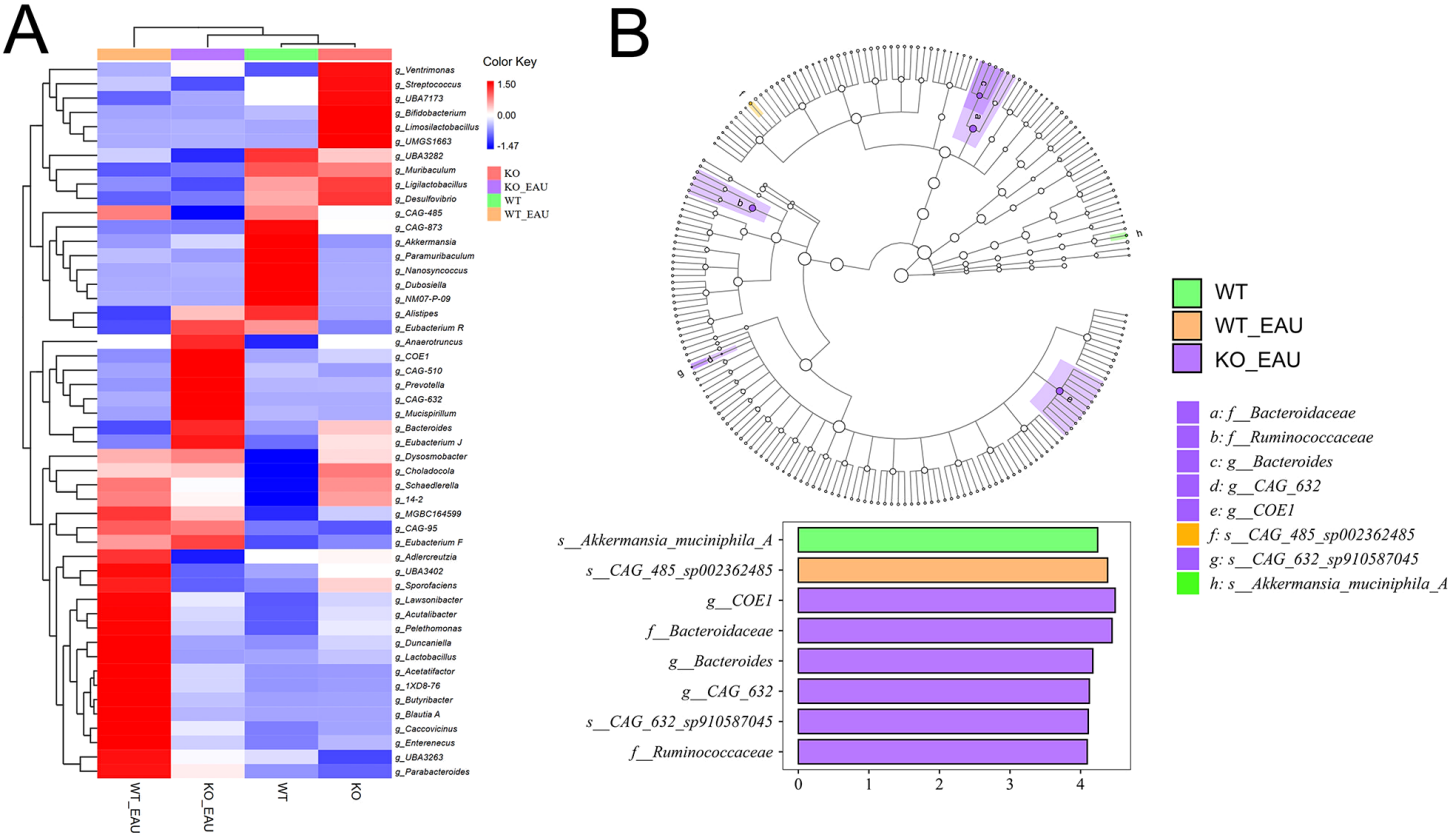
**(A)** The heatmap with hierarchical clustering of gut microbiota abundance at genus level indicated that PD-L1 knockout alone (KO group) caused less variation in microbe distribution than the induction of EAU and EAU combined with PD-L1 knockout. **(B)** LEfSe indicated that EAU could lead to the loss of *Akkermansia muciniphila A* and increased abundance of *CAG-485* sp*002362485*. PD-L1 knockout in EAU could lead to the increased abundance of the family Bacteroidaceae, Lachnospiraceae (*COE1* spp. and *CAG-632* spp.) and Ruminococcaceae. LEfSe, linear discriminant analysis (LDA) effect size; WT group, wild type control group; KO group, PD-L1 knockout group; WT_EAU group, group of EAU induced in wild type mice; KO_EAU group, group of EAU induced in PD-L1 knockout mice.

Microbial composition of gut microbiota was analyzed using hierarchical clustering at the level of genus; PD-L1 knockout alone seemed to cause less variation in the abundance of microbial genera than induction of EAU or EAU combined with PD-L1 knockout (**Figure 5A**). Further comparison via LEfSe analysis showed that induction of EAU could result in a decreased abundance of *Akkermansia muciniphila A* and an increased abundance of *CAG-485* sp*002362485* (member of the family Prevotellaceae), and that PD-L1 knockout in EAU could lead to the increased abundance of Ruminococcaceae family, *COE1* spp. and *CAG-632* spp. (**Figure 5B**).

**Figure 6:**
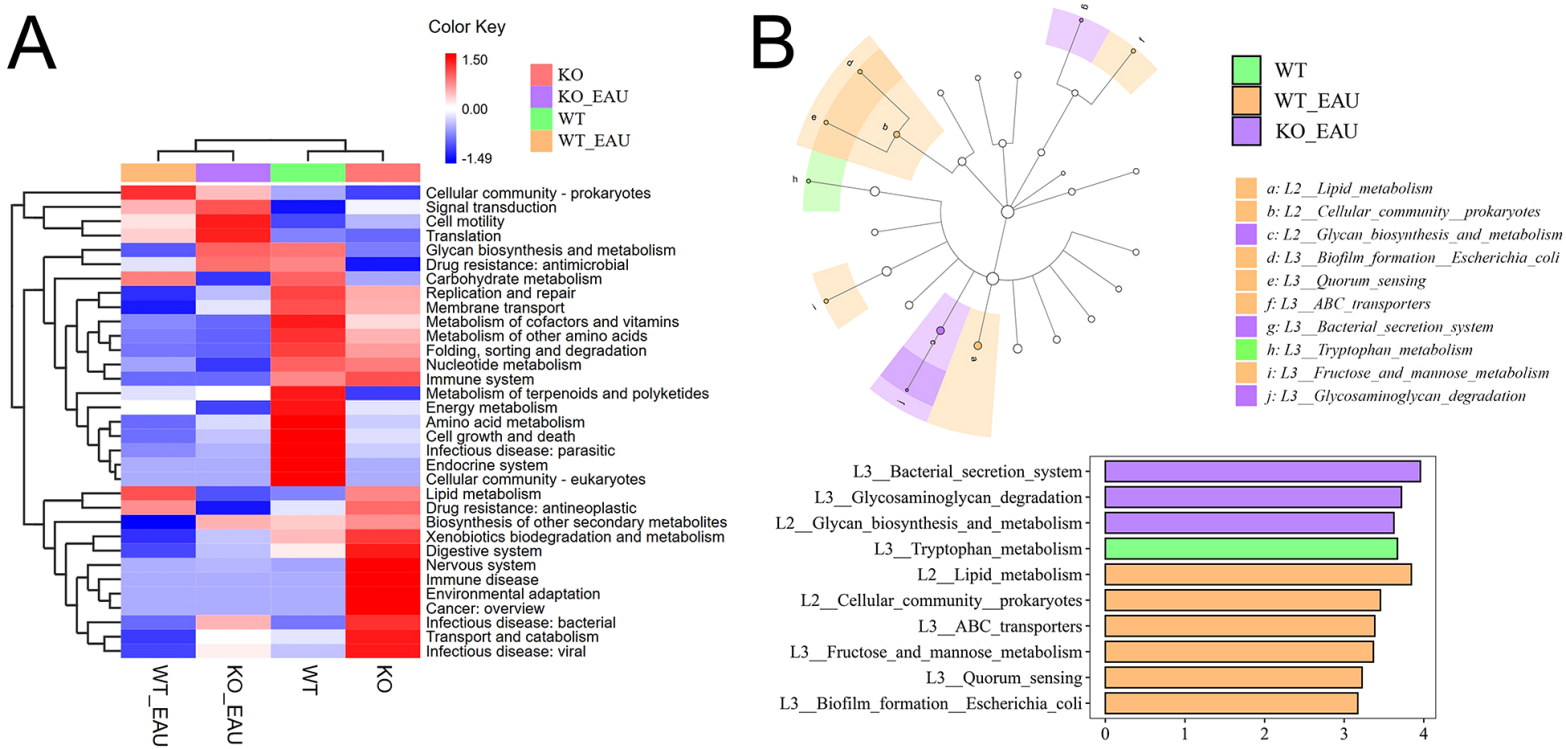
**(A)** The heatmap with hierarchical clustering showed the variations in microbial function annotated by KEGG. **(B)** LEfSe of microbial function indicated that the induction of EAU led to the variations mainly enriched in the pathways of lipid metabolism and carbohydrate metabolism, and that PD-L1 knockout in EAU might further lead to the variation in glycan biosynthesis and metabolism. LEfSe, linear discriminant analysis (LDA) effect size; WT group, wild type control group; KO group, PD-L1 knockout group; WT_EAU group, group of EAU induced in wild type mice; KO_EAU group, group of EAU induced in PD-L1 knockout mice.

**Figure 7:**
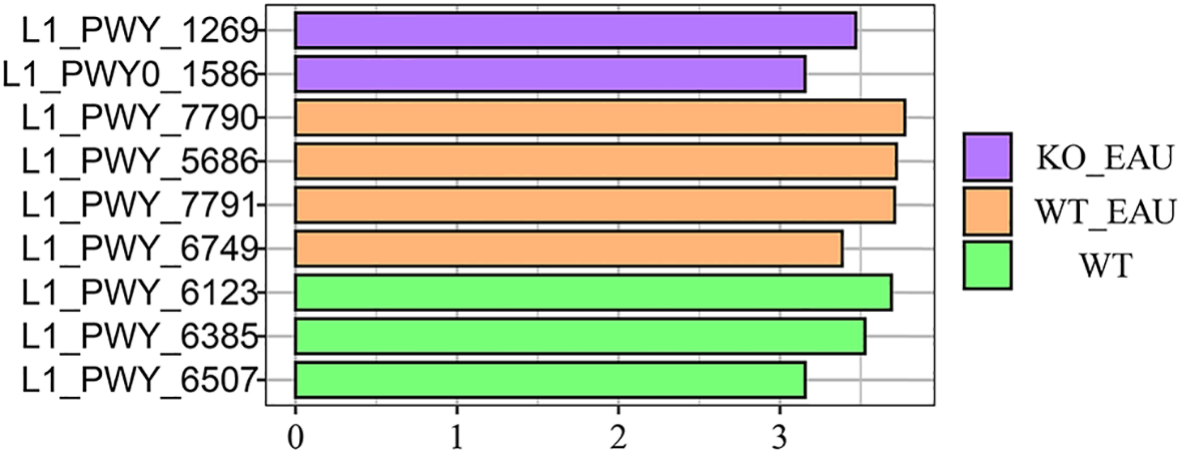
LEfSe of microbial metabolic pathways using annotation of MetaCyc database showed that UMP biosynthesis pathways (PWY-5686, PWY-7790 and PWY-7791) were enriched in EAU mice, and that PD-L1 knockout in EAU mice led to the enrichment of 3-deoxy-α-D-manno-2-octulosonate biosynthesis pathway (PWY-1269) and peptidoglycan maturation pathway (PWY0-1586). LEfSe, linear discriminant analysis (LDA) effect size; WT group, wild type control group; WT_EAU group, group of EAU induced in wild type mice; KO_EAU group, group of EAU induced in PD-L1 knockout mice; UMP, uridine monophosphate.

Hierarchical clustering analysis of functional variations in gut microbiota showed that the induction of EAU had the greatest impact on microbial function (**Figure 6A**). LEfSe analysis with annotation using KEGG database showed that pathways related to tryptophan metabolism were down-regulated in WT_EAU group and KO_EAU group; varied gut microbial functions of WT_EAU group were enriched in the metabolism of lipid, fructose and mannose, while those of KO_EAU group were enriched in the metabolism of glycan and glycosaminoglycan (**Figure 6B**). LEfSe analysis of metabolic pathways using MetaCyc database showed that uridine monophosphate (UMP) biosynthesis pathways (PWY-5686, PWY-7790 and PWY-7791) were enriched in the WT_EAU group, and that 3-deoxy-α-D-manno-2-octulosonate (Kdo) biosynthesis pathway (PWY-1269) and peptidoglycan maturation pathway (PWY0-1586) was enriched in KO_EAU group (**Figure 7**).

## 4 Discussion

In this study, we compared the gut microbiota among wild type mice, PD-L1 knockout mice, wild type mice with EAU and PD-L1 knockout mice with EAU, and preliminarily evaluated potential influences of EAU and PD-L1 on the diversity, composition and function of the gut microbiome. Our results suggest that the induction of EAU exhibited the most significant impact on the richness of gut microbiota, followed by the combination of EAU and PD-L1 knockout.

A previous study on fecal microbiota in patients with autoimmune hepatitis (AIH) showed that AIH was correlated with decreased species richness and evenness (22). Similarly, in our study, the overall richness of gut microbiota in EAU mice significantly decreased when compared to that of control mice, which is in line with findings from AIH. The role of PD-1/PD-L1 in shaping the gut microbiome has been less explored, with few studies focusing on its effect on microbial richness. In this study, PD-L1 knockout was associated with an increase in microbial richness in the context of EAU, although it remains unclear whether the increased richness exacerbates EAU or represents a compensatory response to the absence of PD-L1. Further research involving direct manipulation of microbial richness is required to clarify its role in PD-L1-related autoimmunity.

Both EAU and PD-L1 knockout results in substantial changes in the abundance of specific species or genera within the gut microbiota. Several studies revealed a decreased Firmicutes/Bacteroidetes ratio in fecal microbiota of patients with systemic lupus erythematosus (SLE) and autoimmune thyroid disorders (23, 24). In our study, PD-L1 knockout in EAU mice led to increased disease severity, accompanied by an increased abundance of the Bacteroidaceae family, which mirrors the reduced Firmicutes/Bacteroidetes ratio observed in autoimmune diseases, and might partially explain the aggravated inflammation in PD-L1 knockout mice. Additionally, reduced abundance of *Akkermansia muciniphila A* and increased abundance of the genus *Prevotella* have been associated with autoimmunity (24–28). These changes were also observed in the WT_EAU group and KO_EAU group, suggesting that the abundance of the phylum Bacteroidetes, genus *Akkermansia* and genus *Prevotella* could serve as potential biomarkers and therapeutic targets for EAU. Moreover, PD-L1 knockout led to the increase in *COE1* spp. and *CAG-632* spp. of the family Lachnospiraceae and *Ruminococcus* spp. of the family Ruminococcaceae in EAU model. In another study comparing gut microbiota among various autoimmune diseases (29), a number of members from the genus *Ruminococcus* and family Lachnospiraceae were found to be enriched in diseases without ocular involvement (including ankylosing spondylitis, rheumatoid arthritis, Crohn’s disease and ulcerative colitis) rather than Behçet’s disease (BD) or Vogt-Koyanagi-Harada disease (VKH). This study and our findings implied that PD-L1 might affect uveitis and gut microbiota in a manner different from common etiologies of uveitis. Besides, several genus enriched in BD and VKH (e.g. *Ramularia*, *Alternaria*, *Rhizophagus*, *Bilophila*, *Parabacteroides*, *Paraprevotella*, etc.) (25, 28) were not observed to be differentially abundant among animal models of our study.

Variation in the microbial metabolism has been implicated in numerous autoimmune diseases. In our study, a decline in tryptophan metabolism of the gut microbiota was observed in EAU mice. In experimental autoimmune encephalomyelitis (EAE), deficiency in indoleamine 2,3-dioxygenase (IDO), an enzyme in tryptophan catabolism, led to exacerbated EAE, while administration of tryptophan downstream metabolites could ameliorate EAE by increasing Tregs and inhibiting Th1 and Th17 cells (30). Moreover, we found that knockout of PD-L1 in EAU mice led to additional enrichment in the microbial biosynthesis of Kdo. Kdo is the core component of lipopolysaccharide (LPS) and is essential for the viability and endotoxin activity of most Gram-negative bacteria (31). Therefore, the enhanced Kdo synthesis of gut microbiota might participate in the aggravation of EAU induced by PD-L1 KO. While these findings suggest an association among EAU, PD-L1, and microbial metabolism, further investigation is required to understand the precise relationships among these factors. Further studies combining gut microbiota profiling with metabolomics will be critical to addressing these questions.

A limitation of our study was the relatively small number of biological replicates, which may have constrained the identification of additional microbial species or functional pathway. Expanding the number of replicates might enhance the robustness of our findings. Additionally, while this study highlights correlations among EAU, PD-L1, and the gut microbiota, establishing a clear cause-and-effect remains challenging. Future experiments using interventions including antibiotics targeting certain taxa, dietary modifications or fecal transplantation could provide deeper insights into these interactions; *in vitro* and *in vivo* validation of the relation among microbial metabolites, T cell responses and PD-L1 signaling should be performed.

In conclusion, our findings indicate that PD-L1 knockout in EAU exacerbates inflammation and alters the richness, composition and metabolic function of gut microbiota. Members of the family Bacteroidaceae, family Lachnospiraceae, genus *Akkermansia*, genus *CAG-485* and genus *Ruminococcus* could serve as potential biomarkers for EAU severity and response for PD-1-related therapies.

## Data Availability

The data presented in the study are deposited in the Sequence Read Archive (SRA) repository, BioProject ID: PRJNA1261602 (http://www.ncbi.nlm.nih.gov/sra/PRJNA1261602).
